# Targeting TBK1 Attenuates LPS-Induced NLRP3 Inflammasome Activation by Regulating of mTORC1 Pathways in Trophoblasts

**DOI:** 10.3389/fimmu.2021.743700

**Published:** 2021-11-09

**Authors:** Sohee Lee, Jiha Shin, Jong-Seok Kim, Jongdae Shin, Sung Ki Lee, Hwan-Woo Park

**Affiliations:** ^1^ Department of Cell Biology, Konyang University College of Medicine, Daejeon, South Korea; ^2^ Myunggok Medical Research Institute, Konyang University College of Medicine, Daejeon, South Korea; ^3^ Department of Obstetrics and Gynecology, Konyang University Hospital, Daejeon, South Korea

**Keywords:** TBK1, NLRP3 inflammasome, maternal inflammation, trophoblast, placenta, mTORC1

## Abstract

Pathological maternal inflammation and abnormal placentation contribute to several pregnancy-related disorders, including preterm birth, intrauterine growth restriction, and preeclampsia. TANK-binding kinase 1 (TBK1), a serine/threonine kinase, has been implicated in the regulation of various physiological processes, including innate immune response, autophagy, and cell growth. However, the relevance of TBK1 in the placental pro-inflammatory environment has not been investigated. In this study, we assessed the effect of TBK1 inhibition on lipopolysaccharide (LPS)-induced NLRP3 inflammasome activation and its underlying mechanisms in human trophoblast cell lines and mouse placenta. TBK1 phosphorylation was upregulated in the trophoblasts and placenta in response to LPS. Pharmacological and genetic inhibition of TBK1 in trophoblasts ameliorated LPS-induced NLRP3 inflammasome activation, placental inflammation, and subsequent interleukin (IL)-1 production. Moreover, maternal administration of amlexanox, a TBK1 inhibitor, reversed LPS-induced adverse pregnancy outcomes. Notably, TBK1 inhibition prevented LPS-induced NLRP3 inflammasome activation by targeting the mammalian target of rapamycin complex 1 (mTORC1). Thus, this study provides evidence for the biological significance of TBK1 in placental inflammation, suggesting that amlexanox may be a potential therapeutic candidate for treating inflammation-associated pregnancy-related complications.

## Introduction

During pregnancy, at the maternal-fetal interface, physiological inflammation is beneficial for the implantation and the preliminary stages of placentation. An adequate response of the immune system at the placenta is vital to protect the fetus from various stimuli, such as infection and pathogens (1, 2). However, pathological inflammatory conditions can cause placental insufficiency, thereby contributing to several pregnancy-related disorders, including preterm birth, intrauterine growth restriction, and preeclampsia (3–5). Trophoblasts in the placenta recognize and respond to pathogen-associated molecular patterns such as bacterial cell wall components through the expression of toll-like receptors (TLRs) (6). Activation of TLRs stimulates intracellular signaling cascade, resulting in the activation of nuclear factor-kappa B (NF-κB/p65) and expression of pro-inflammatory cytokines, including interleukin (IL)-1, IL-6, and tumor necrosis factor (TNF)-α, which contribute to trophoblast dysfunction and placental abnormalities (7–9).

The NLR family pyrin domain-containing 3 (NLRP3) inflammasome is a large cytosolic multiprotein complex composed of an NLRP3 scaffold, an apoptosis-associated speck-like protein (ASC) adaptor, and caspase-1 as an IL-1β-converting enzyme (10). They are activated by a wide range of pathogen-associated molecular patterns, such as a TLR4 ligand, lipopolysaccharide (LPS), or damage-associated molecular patterns such as ATP, and play a role in inflammation (11). In response to stimuli, NLRP3 inflammasomes mediate the activation of caspase-1 and cleavage of inactive pro-IL-1β into its active form (12, 13) and are related to pregnancy dysfunction, including preterm birth, fetal growth restriction, and preeclampsia (14–16). Aberrant IL-1β production in the placenta and human trophoblasts has been associated with NLRP3 inflammasome activation, contributing to placental inflammation (17).

TANK-binding kinase 1 (TBK1) is a non-canonical IKK‐related innate immune kinase that plays a pivotal role in the innate immune system (18, 19). TBK1 can be activated by phosphorylation in response to stimulation by TLR3, TLR4, and pathogens (20); however, the molecular mechanisms involved in the phosphorylation of TBK1 are not entirely understood. Our previous study showed that activated TBK1 induces obesity-induced ubiquitinated protein inclusions in liver tissue and that inhibiting the TBK1 pathway protects against fibrotic liver pathologies in mouse models of obesity and nonalcoholic steatohepatitis (21). TBK1 has been associated with stimulus-dependent NF-κB activation, affecting several diseases, including inflammatory disorders and cancer (22–24). However, the relevance of TBK1 in LPS-induced NLRP3 inflammasome activation in the placenta and trophoblasts remains unexplored.

In this study, the potential effect of TBK1 inhibition on NLRP3 inflammasome activation in LPS-treated pregnant mice and human trophoblast cell lines was investigated for the first time. Further, the molecular mechanisms by which TBK1 regulates LPS-induced NLRP3 inflammasome activation was also studied. Our results suggest that TBK1 phosphorylation is induced in the placental tissues of LPS-treated mice and LPS-treated trophoblasts. Both pharmacological inhibition and genetic knockdown of TBK1 ameliorated LPS-induced NLRP3 inflammasome activation and placental inflammation. Moreover, we found that TBK1 deficiency suppresses LPS-induced NLRP3 inflammasome activation *via* a mTORC1-dependent mechanism. Therefore, TBK1 inhibition may act as a potential therapeutic target for placental dysfunction associated with NLRP3 inflammasome and inflammation.

## Materials and Methods

### Reagents

Immunoblotting was performed using antibodies against phospho-TBK1, TBK1, NLRP3, Caspase-1, phospho-p65, p65, phospho-IκB, IκB, phospho-S6K, S6K (Cell Signaling Technology, Danvers, MA, USA), Caspase-1 (Santa Cruz Biotechnology, Dallas, TX, USA), and α-tubulin (Developmental Studies Hybridoma Bank, Iowa City, IA, USA). LPS (*Escherichia coli* LPS, serotype 0127: B8), ATP, and rapamycin were purchased from Sigma-Aldrich (St. Louis, MO, USA). Amlexanox and Torin 1 were purchased from the Cayman Chemical Company, Ann Arbor, MI, USA. The sources of other reagents are indicated in the specified methods.

### Animals and Treatments

Female C57BL/6 mice (6–8-weeks-old) were purchased from Samtako (Osan, Korea). All mice were maintained under a controlled temperature (20–25°C) and humidity (50 ± 5%) with a 12 h light/dark cycle and free access to food and drinking water. Female mice were mated with male mice, and the presence of vaginal plugs the following day confirmed successful mating. The timed pregnant mice received an intraperitoneal injection of LPS (200 µg/kg) or saline on gestation day 17.5. In the amlexanox + LPS group, the pregnant mice were intraperitoneally injected with amlexanox (2.5 mg/kg) at 27 h and 3 h before the intraperitoneal administration of LPS. The pregnant mice were sacrificed at various time points (4, 6, 8, 10, or 12 h) after LPS treatment, and placental samples were collected for molecular analysis. All animal studies were conducted following the Guidelines for the Care and Use of Laboratory Animals of the National Institutes of Health and approved by the Animal Ethics Committee of Konyang University (P-21-19-A-01).

### Cell Culture and Treatments

Human first-trimester trophoblast cell lines, HTR-8/SVneo (American Type Culture Collection, Rockville, MD, USA) and Sw.71 (gifted by Dr. Gil Mor, Yale University School of Medicine, New Haven, CT, USA), were used in our experiments. HTR-8/SVneo cells were cultured in RPMI 1640 medium (Welgene, Gyeongsan, Korea) supplemented with 10% fetal bovine serum (Welgene), 4500 mg/L D-glucose, 10 mM HEPES, 2mM L-glutamine, 1 mM sodium pyruvate, and 100 U/mL penicillin-streptomycin (Welgene). The Sw.71 cells were cultured in Dulbecco’s modified Eagle’s medium (Welgene) containing 10% fetal bovine serum, 4500 mg/L D-glucose with L-glutamine, and 100 U/mL penicillin-streptomycin. All cultures were maintained in a humidified 5% CO_2_ atmosphere at 37°C. For LPS treatment, cells were incubated in the presence of LPS or treated with LPS and then treated with 5 mM ATP for 45 min, as described previously (17). Identical volumes of phosphate-buffered saline (PBS) were used as the vehicle controls. When indicated, the cells were incubated with the TBK1 inhibitor amlexanox.

### Cytotoxicity Assay

The HTR-8/SVneo cell survival was measured using the WST-1 assay (EZ-CytoX Enhanced Cell Viability Assay Kit, Daeil Lab Service, Seoul, Korea) according to the manufacturer’s protocol (25). Briefly, the HTR-8/SVneo cells (1 × 10^4^ cells/well) were plated into 96-well plates and treated with the indicated concentrations of amlexanox for 24 h. Subsequently, 10 μL of WST-1 reagent was added to each well and incubated for 30 min at 37°C in a 5% CO_2_ incubator (Thermo Scientific, Waltham, MA, USA). The absorbance was measured at 450 nm using an Epoch 2 microplate reader (Bio-Tek Instruments, Winooski, VT, USA), and the absorbance values of the treated cells were expressed as a percentage of the absorbance values of the control.

### Plasmids and Viral Production

Using a polyethylenimine reagent, HEK293T cells were transfected with the following lentiviral constructs with the packaging plasmids: sh-Luciferase (sh-Luc) and sh-TBK1 constructs (obtained from the RNAi Consortium collection of the Broad Institute). Lentiviral supernatants were collected and filtered 48 and 72 h after transfection. HTR-8/SVneo cells and Sw.71 cells were incubated 2–3 d with the lentiviral medium in the presence of 4 μg/mL polybrene.

### Quantitative Real-Time PCR

Total RNA was extracted from placental tissues and HTR-8/SVneo cells using TRIzol reagent (Takara, Shiga, Japan) according to the manufacturer’s instructions. Complementary DNA was synthesized from RNA using a cDNA synthesis kit (BioFact, Daejeon, Korea). Quantitative real-time reverse transcription PCR (qRT-PCR) was performed in triplicate with the SYBR Green Real-Time PCR Master Mix Reagent (BioFact) using the QuantStudio 3 Real-time PCR System (Life Technologies, Carlsbad, CA, USA). Relative mRNA expression was calculated using comparative threshold cycle (C_t_) values normalized to those of mouse or human cyclophilin. The following primers were used: mouse *Nlrp3*: forward 5′- AGCCTTCCAGGATCCTCTTC-3′, reverse 5′-CTTGGGCAGCAGTTTCTTTC-3′; mouse *Tnf-α*: forward 5′-TCCCAGGTTCTCTTCAAGGGA-3′, reverse 5′-GGTGAGGAGCACGTAGTCGG-3′; mouse *Tgf-β1*: forward 5′-CTCCCGTGGCTTCTAGTGC-3′, reverse 5′-GCCTTAGTTTGGACAGGATCTG-3′; mouse *Il-6*: forward 5′- TAGTCCTTCCTACCCCAATTTCC-3′, reverse 5′-TTGGTCCTTAGCCACTCCTTC-3′; mouse *Il-10*: forward 5′-GCTCTTACTGACTGGCATGAG-3′, reverse 5′-CGCAGCTCTAGGAGCATGTG-3′; mouse *Mcp-1*: forward 5′-CATCCACGTGTTGGCTCA-3′, reverse 5′-GATCATCTTGCTGGTGAATGAGT-3′; mouse *Il-1β*: forward 5′-TCTTTGAAGTTGACGGACCC-3′, reverse 5′-TGAGTGATACTGCCTGCCTG-3′; mouse *IFN-β*: forward 5′-GCACTGGGTGGAATGAGACT-3′, reverse 5′-AGTGGAGAGCAGTTGAGGAC-3′; mouse *Cyclophilin A*: forward 5′-GAGCTGTTTGCAGACAAAGTTC-3′, reverse 5′-CCCTGGCACATGAATCCTGG-3′; human *NLRP3*: forward 5′-GATCTTCGCTGCGATCAACAG-3′, reverse 5′-CGTGCATTATCTGAACCCCAC-3′; human *IL-1β*: forward 5′-AAAGAGGCACTGGCAGAA-3′, reverse 5′-AGCTCTGGCTTGTTCCTCAC-3′; and human *Cyclophilin A*: forward 5′-GCAAAGTGAAAGAAGGCATGAA-3′, reverse 5′-CCATTCCTGGACCCAAAGC-3′.

### Immunoblotting

Placental tissues, HTR-8/SVneo cells, and Sw.71 cells were lysed in an ice-cold radioimmunoprecipitation assay buffer containing cOmplete protease inhibitor cocktail (Roche, Basel, Switzerland). Lysates were incubated for 20 min on ice and centrifuged at 18,000 *× g* for 15 min at 4°C. Protein concentrations were measured using a bicinchoninic acid protein assay (Pierce, Rockford, IL, USA). Lysates were boiled in 1× sodium dodecyl sulfate (SDS) and Laemmli sample buffer for 5 min. Proteins were separated by SDS-polyacrylamide gel electrophoresis and transferred to polyvinylidene fluoride membranes (Millipore, Burlington, MA, USA), which were probed with primary antibodies against p-TBK1, TBK1, NLRP3, caspase-1, p-p65, p65, p-IκBα, IκBα, p-S6K, S6K, and α-tubulin. After incubation with secondary antibodies conjugated with horseradish peroxidase (Bio-Rad, Hercules, CA, USA), chemiluminescence was detected using a Fusion Solo System (Vilber Lourmat, Marne-la-Vallée, France). Densitometric analysis of the blots was performed using ImageJ (National Institutes of Health, Bethesda, MD, USA), in which the background was removed for each band.

### Histology

Mouse placental tissues were fixed in 10% neutral buffered formalin for 24 h, dehydrated, and embedded in paraffin. Sections (5 μm) of the embedded tissue were prepared and stained with hematoxylin and eosin dyes or periodic acid-Schiff dyes. For immunohistochemistry, the paraffin-embedded sections were deparaffinized, rehydrated, and treated for antigen retrieval. Endogenous peroxidase was quenched using 3% hydrogen peroxide. After blocking the nonspecific antigens, the sections were incubated with anti-NLRP3 antibody or anti-IL-1β antibody overnight at 4°C, followed by incubation with biotinylated secondary antibody (Vector Laboratories, Burlingame, CA, USA). Antibodies were visualized with streptavidin-HRP (BD Pharmingen, San Diego, CA, USA) using diaminobenzidine (Sigma-Aldrich). Hematoxylin was used to visualize nuclei. The samples were analyzed under a light microscope (DM2500; Leica, Wetzlar, Germany).

### Enzyme-Linked Immunosorbent Assay (ELISA)

Cell supernatants collected from the HTR-8/SVneo cells were assayed for the levels of the secreted inflammatory cytokine IL-1β using the DuoSet ELISA development kit (R&D Systems, Minneapolis, MN, USA) according to the manufacturer’s instructions. The optical density of the final colored reaction product was determined at 450 nm using an Epoch 2 microplate reader (Bio-Tek Instruments).

### Statistical Analysis

Results are presented as the mean ± standard error of the mean (SEM). Data presented in the figures are representative of at least three independent experiments unless stated otherwise. The significance of differences between the two experimental groups was determined using a two-tailed Student’s *t*-test. Multiple comparisons were conducted using one-way ANOVA followed by Tukey’s or Fisher’s least significant difference (LSD) *post hoc* test. Differences were considered statistically significant at *p* < 0.05, *p < 0.05, **p < 0.01, ***p < 0.001).

## Results

### TBK1 Phosphorylation Is Induced in the Placentas and Trophoblast Cells After LPS Exposure

**Figure 1:**
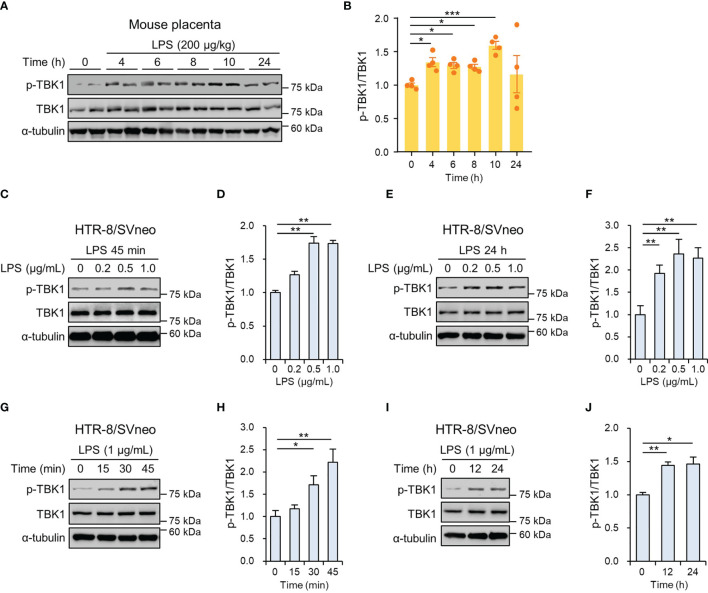
TBK1 phosphorylation in the placenta of mice and human trophoblasts exposed to lipopolysaccharide (LPS). **(A, B)** Pregnant mice were injected intraperitoneally with LPS (200 μg/kg) on gestation day 17.5. After the indicated number of hours, placental tissues were collected from mice and analyzed by immunoblotting with anti-p-TBK1 and TBK1 antibodies (*n* = 4 per group). **(C–F)** HTR-8/SVneo cells were treated with the indicated concentration of LPS for 45 min **(C, D)** and 24 h **(E, F)**. Cell lysates were analyzed by immunoblotting with anti-p-TBK1 and anti-TBK1 antibodies. **(G–J)** HTR-8/SVneo cells were treated with LPS (1 μg/mL) for the indicated periods. Cell lysates were analyzed by immunoblotting with anti-p-TBK1 and anti-TBK1 antibodies. α-tubulin served as a loading control. Band intensities were quantified and normalized to total protein intensities. Data are shown as means ± SEM. Results are representative of at least three independent experiments. *p < 0.05; **p < 0.01; ***p < 0.001 (Student’s *t*-test).

We evaluated the phosphorylation of TBK1 protein in the mouse placenta by LPS-induced maternal inflammatory response. Immunoblot analysis showed that the placental TBK1 phosphorylation was significantly increased at 4, 6, 8, and 10 h after LPS treatment (**Figures 1A, B**). To measure the phosphorylated levels of TBK1 in trophoblasts during maternal inflammation, human first-trimester extravillous trophoblasts (HTR-8/SVneo and Sw.71) were stimulated with various LPS concentrations at various time points. Immunoblot analysis showed that LPS increased TBK1 phosphorylation in HTR-8/SVneo after both short- (**Figures 1C, D**) and long-term treatments (**Figures 1E, F**) in a dose-dependent manner. The time course of TBK1 phosphorylation was further investigated by treating the cells with LPS (1 μg/mL), and the results showed that short- and long-term exposure to LPS significantly increased phosphorylated TBK1 levels in HTR-8/SVneo cells in a time-dependent manner (**Figures 1G–J**). Similar to that in HTR-8/SVneo cells, the expression of phosphorylated TBK1 was stimulated by LPS treatment in Sw.71 cells (**Supplemental Figure 1**).

### TBK1 Deficiency Attenuates LPS-Induced NLRP3 Inflammasome Activation in Trophoblasts

**Figure 2:**
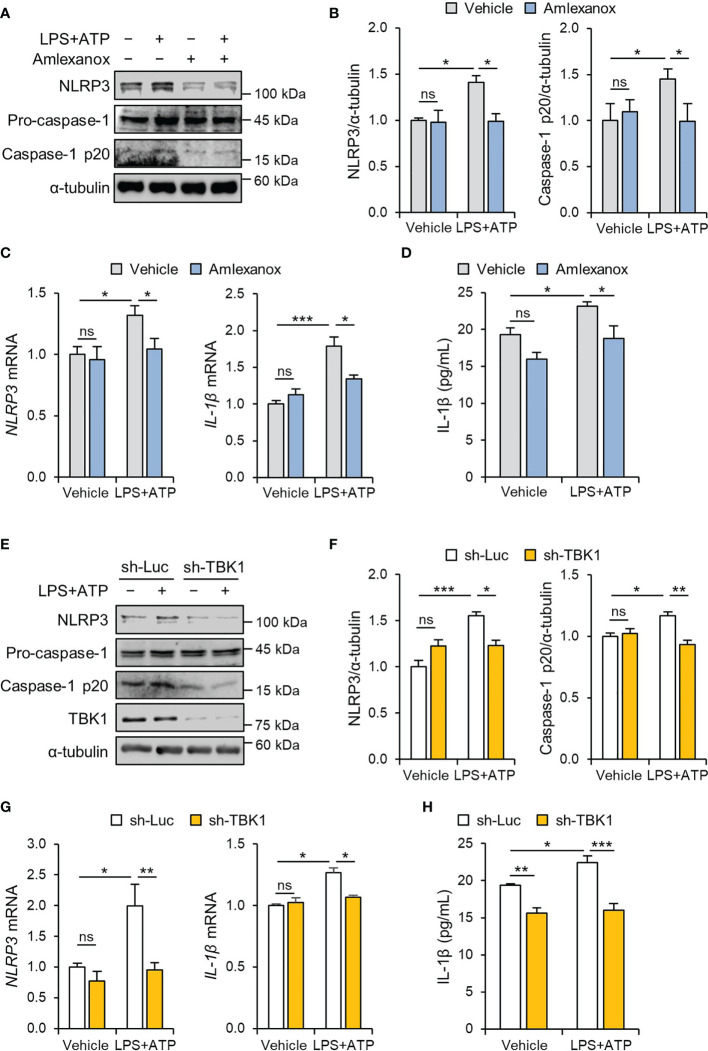
TBK1 deficiency abolishes LPS-induced NLRP3 inflammasome activation in trophoblasts. **(A–C)** HTR-8/SVneo cells were treated with 1 μg/mL LPS or 20 µM amlexanox for 24 h **(A, B)** or 12 h **(C)** followed by treatment with 5 mM ATP for 45 min, as indicated in the figure. **(A, B)** Cell lysates were analyzed by immunoblotting with anti-NLRP3 and anti-caspase-1 antibodies. **(C)** Relative mRNA expression of NLRP3 and IL-1β was quantified by qRT-PCR. **(D)** Conditioned media were collected from HTR-8/SVneo cells treated with 1 μg/mL LPS or 10 µM amlexanox for 36 h followed by treatment with 5 mM ATP for 45 min, as indicated in the figure. IL-1β levels were quantified by ELISA. **(E–G)** HTR-8/SVneo cells were infected with lentiviruses expressing shRNAs targeting luciferase (sh-Luc) or TBK1 (sh-TBK1) and treated with 1 μg/mL LPS for 24 h **(E, F)** or 12 h **(G)** followed by treatment with 5 mM ATP for 45 min, as indicated in the figure. **(E–G)** Cell lysates were analyzed by immunoblotting with anti-NLRP3, anti-caspase-1, and anti-TBK1 antibodies. **(G)** Relative mRNA expression of NLRP3 and IL-1β was quantified by qRT-PCR. α-tubulin served as a loading control. Band intensities were quantified and normalized to control band intensities. **(H)** Conditioned media were collected from HTR-8/SVneo cells infected with sh-Luc or sh-TBK1 lentiviruses and treated with 1 μg/mL LPS for 24 h followed by treatment with 5 mM ATP for 45 min, as indicated in the figure. IL-1β levels were quantified by ELISA. Data are shown as means ± SEM. Results are representative of at least three independent experiments. *p < 0.05; **p < 0.01; ***p < 0.001; ns, not significant (One-way ANOVA, followed by Tukey’s test).

NLRP3 inflammasome activation in trophoblasts is implicated in the pathogenesis of placental inflammation (17, 26). To investigate the effects of LPS on placental NLRP3 protein expression, placentas from mice exposed to LPS were analyzed by immunohistochemistry. The results showed that NLRP3 protein expression was increased in trophoblasts in the junctional zone and the labyrinth of the placentas of the LPS-treated mice compared with that of the control placentas of saline-treated mice (**Supplemental Figure 2**). Consistent with the above results, the NLRP3 inflammasome was activated in HTR-8/SVneo and Sw.71 cells upon stimulation with LPS and ATP (**Supplemental Figures 3A, B**). Furthermore, we investigated whether TBK1 is involved in LPS-induced NLRP3 inflammasome activation. Amlexanox, a TBK1 inhibitor, was used to explore the effect of TBK1 deficiency on LPS-induced NLRP3 inflammasome. We measured the cell viability of HTR-8/SVneo and Sw.71 cells treated with different concentrations of amlexanox. *In vitro* cytotoxicity assays revealed that amlexanox exhibited low cytotoxicity against cells up to 200 µM (**Supplemental Figures 4A, B**). Immunoblot analysis showed that amlexanox markedly decreased LPS-induced NLRP3 mRNA levels and NLRP3 and cleaved caspase-1 protein levels (**Figures 2A–C** and **Supplemental Figure 3C**). To confirm these results, IL-1β levels were analyzed using ELISA. Pretreatment of HTR-8/SVneo cells with amlexanox resulted in significantly lower levels of IL-1β compared to that in LPS-treated cells (**Figure 2D**).

To clarify the specific signaling mechanism by which amlexanox suppresses LPS-induced NLRP3 inflammasome activation in trophoblasts, lentiviral vectors expressing small hairpin RNAs (shRNAs) targeting human TBK1 were transduced to HTR-8/SVneo and Sw.71 cells. Knockdown of TBK1 was sufficient to block the upregulation of NLRP3 and cleaved caspase-1 in LPS-treated cells (**Figures 2E–G** and **Supplemental Figure 4D**), consistent with the inhibitory effect of amlexanox on NLRP3 inflammasome activation. ELISA showed that the lentiviral knockdown of TBK1 also decreased IL-1β production (**Figure 2H**), suggesting that it is an inhibitory effect of TBK1, not a nonspecific effect of amlexanox.

### TBK1 Deficiency Attenuates the LPS-Induced NF-κB/p65 Pathway in Trophoblasts

**Figure 3:**
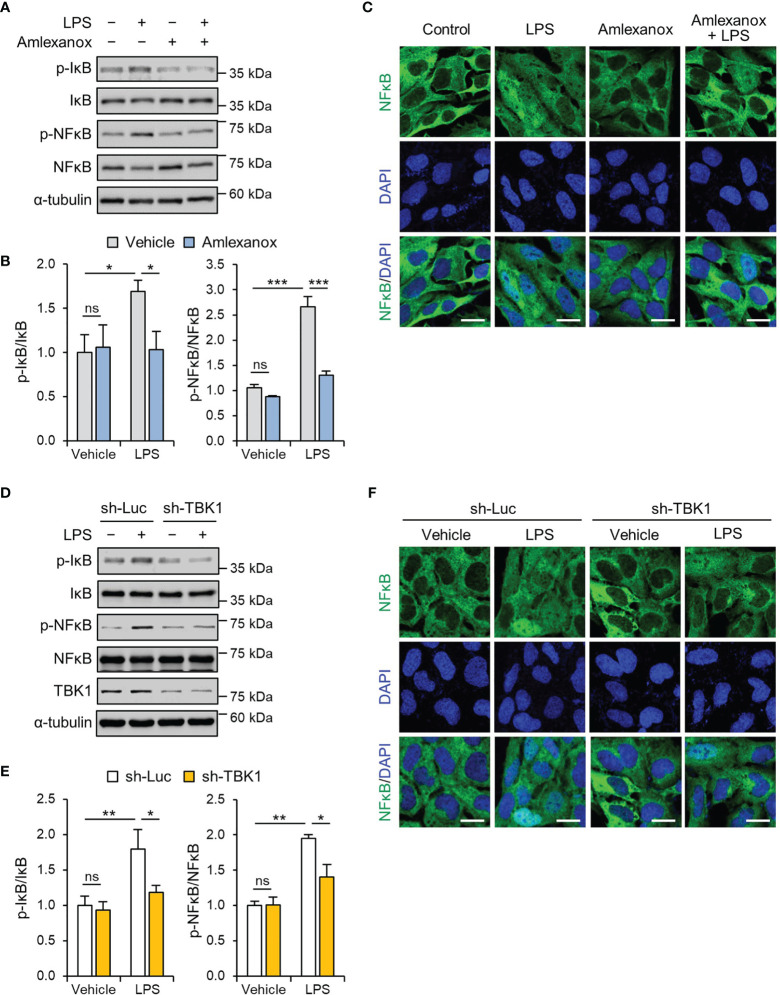
TBK1 deficiency abolishes LPS-induced NF-κB/p65 activation in trophoblasts. **(A, B)** HTR-8/SVneo cells were treated with 1 μg/mL LPS or 100 µM amlexanox for 1 h, as indicated in the figure. Cell lysates were analyzed by immunoblotting with anti-p-IκBα, anti-IκBα, anti-p-p65, and anti-p65 antibodies. α-tubulin served as a loading control. **(C)** Immunofluorescence staining of p65 (green) in HTR-8/SVneo cells treated with 1 μg/mL LPS or vehicle for 45 min in the presence or absence of 100 µM amlexanox. Nuclei were stained with DAPI (blue). Scale bars, 20 μm. **(D, F)** HTR-8/SVneo cells were infected with lentiviruses expressing shRNAs targeting luciferase (sh-Luc) or TBK1 (sh-TBK1) and treated with 1 μg/mL LPS for 1 h, as indicated in the figure. Cell lysates were analyzed by immunoblotting with anti-p-IκBα, anti-IκBα, anti-p-p65, and anti-p65 antibodies. α-tubulin served as a loading control. **(E)** Immunofluorescence staining of p65 (green) in HTR-8/SVneo cells infected with sh-Luc or sh-TBK1 lentiviruses and treated with 1 μg/mL LPS or vehicle for 45 min. Nuclei were stained with DAPI (blue). Scale bars, 20 μm. Data are shown as means ± SEM. Results are representative of at least three independent experiments. *p < 0.05; **p < 0.01; ***p < 0.001; ns, not significant (One-way ANOVA, followed by Tukey’s test).

NF-κB signaling mediates the priming signal of NLRP3 inflammasome activation and upregulates the expression levels of NLRP3 and pro-IL-1β (27, 28). LPS increased the phosphorylation of NF-κB/p65 in a time-dependent manner, and the maximal effect was observed at 1 h following 1 μg/mL LPS treatment (**Supplemental Figure 5A**). To investigate the effect of amlexanox on the NF-κB pathway, we measured the phosphorylation levels of IκBα and NF-κB/p65 in HTR-8/SVneo and Sw.71 cells. Pretreatment with amlexanox decreased LPS-induced phosphorylation of IκBα and NF-κB/p65 (**Figures 3A, B**, and **Supplemental Figure 5B**). Further, we investigated the nuclear translocation of NF-κB/p65. LPS treatment resulted in a significant increase in NF-κB/p65 in the nucleus of HTR-8/SVneo cells, whereas the nuclear translocation of NF-κB/p65 was suppressed by amlexanox (**Figure 3C**).

We also examined whether the knockdown of TBK1 reversed the effect of LPS on the NF-κB/p65 pathway. Lentiviral knockdown of TBK1 reduced LPS-induced phosphorylation of IκBα and NF-κB/p65 (**Figures 3D, E**, and **Supplemental Figure 4C**). Immunofluorescence staining showed that the knockdown of TBK1 was sufficient to inhibit the nuclear translocation of NF-κB/p65 in LPS-treated cells (**Figure 3F**). These results collectively indicate that TBK1 deficiency prevents the LPS-induced nuclear translocation of NF-κB/p65 by phosphorylation of IκBα.

### Amlexanox Alleviates Adverse Pregnancy Outcomes in LPS-Treated Mice

**Figure 4:**
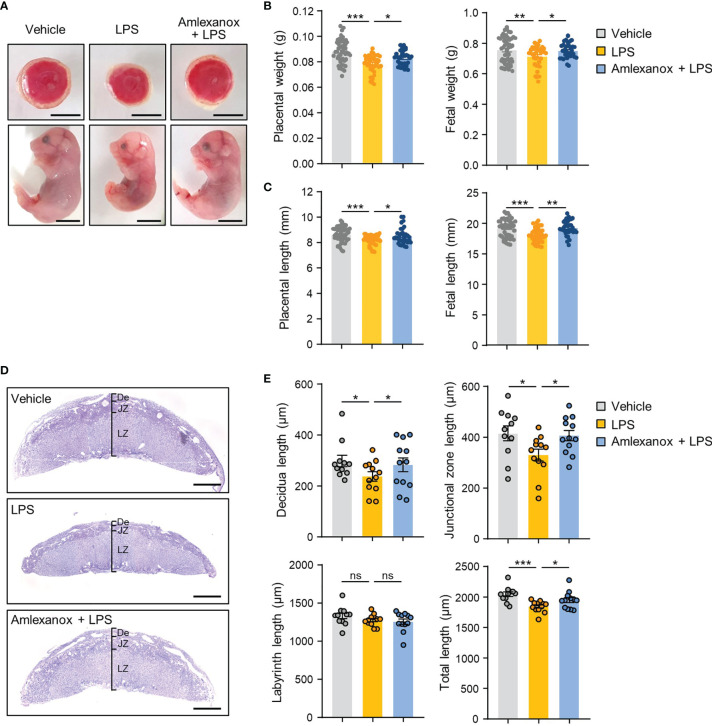
The effect of amlexanox on the gross placental morphology in the LPS-treated pregnant mice. **(A–E)** Pregnant mice were intraperitoneally injected with LPS (200 μg/kg) or amlexanox (2.5 mg/kg) on gestation day 17.5, as indicated in the figure. **(A)** Representative images of fetus and placenta for each maternal group. Scale bars, 0.5 cm **(B, C)** Average fetal and placental weights and lengths for each group (*n* = 33-50 per group). **(D, E)** Periodic acid-Schiff (PAS) staining of placental tissues from vehicle-, LPS-, LPS+amlexanox-treated pregnant mice at gestation day 17.5. Decidua (De), labyrinth (LZ), and junctional zone (JZ) length on the midline section was quantified (*n* = 11 per group). Scale bars, 1 mm. Data are shown as means ± SEM. *p < 0.05; **p < 0.01; ***p < 0.001; ns, not significant (One-way ANOVA, followed by Fisher’s LSD test).

To evaluate whether maternal administration of amlexanox affects the fetal-placental development of LPS-treated mice, we first measured the fetal and placental weight and length in each group. Fetal and placental weight and length were significantly reduced in the LPS-treated mice compared to that in the control mice, whereas amlexanox treatment prevented the decrease in fetal and placental weight and length associated with LPS treatment (**Figures 4A–C**). We also evaluated whether amlexanox affects placental growth and morphology in LPS-treated mice. Periodic acid-Schiff staining revealed that LPS-exposed placenta had significantly reduced length of decidual layers and junctional zones; however, no appreciable change was observed in the length of the labyrinth layer. Interestingly, amlexanox treatment abrogated the LPS-induced placental morphological changes (**Figures 4D, E**). These findings suggest that maternal administration of amlexanox alleviates LPS-induced morphological changes in the placenta.

### Amlexanox Reverses Placental Inflammation and NLRP3 Inflammasome Activation in LPS-Treated Mice

**Figure 5:**
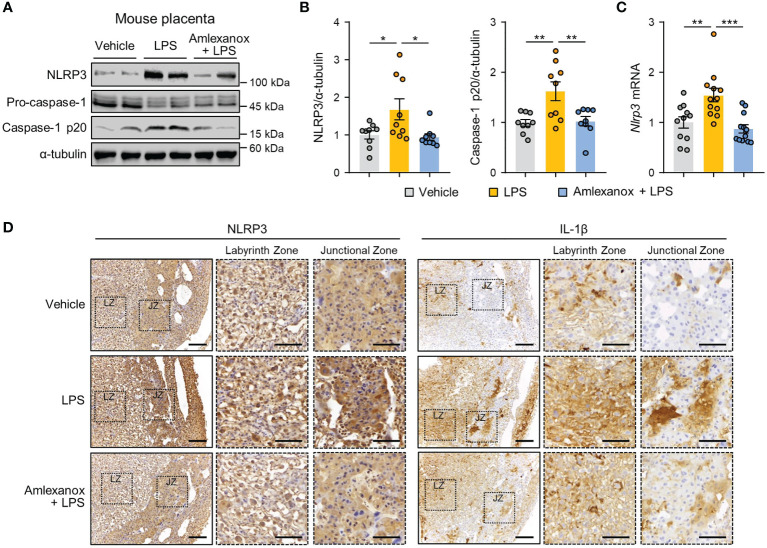
Amlexanox ameliorates placental NLRP3 inflammasome activation in LPS-treated pregnant mice. **(A–D)** Pregnant mice were intraperitoneally injected with LPS (200 μg/kg) or amlexanox (2.5 mg/kg) on gestation day 17.5, as indicated in the figure. **(A, B)** Placental tissues were collected from mice and analyzed by immunoblotting with anti-NLRP3 and anti-caspase-1 antibodies (*n* = 9 per group). α-tubulin served as a loading control. Band intensities were quantified and normalized to control band intensities. **(C)** Placental tissues were collected from mice and analyzed by qRT-PCR for the expression of NLRP3 (*n* = 11–12 per group). **(D)** Immunohistochemical analysis of NLRP3 and IL-1β in placental tissues from vehicle-, LPS-, and LPS+amlexanox-treated pregnant mice at gestation day 17.5. Nuclei were stained with hematoxylin. Boxed areas are magnified in the adjacent panels. LZ and JZ represent the labyrinth zone and the junctional zone, respectively. Scale bars, 200 μm; inset, 100 μm. Data are shown as means ± SEM. *p < 0.05; **p < 0.01; ***p < 0.001 (One-way ANOVA, followed by Tukey’s test).

To further confirm whether maternal administration of amlexanox influences placental NLRP3 inflammasome activation *in vivo*, we measured the expression levels of NLRP3 inflammasome markers in the placentas of LPS-treated mice. Immunoblot analysis showed that NLRP3 and cleaved caspase-1 protein levels were elevated in response to LPS treatment; this increase was suppressed in amlexanox-treated mice exposed to LPS (**Figures 5A, B**). Correspondingly, amlexanox treatment decreased NLRP3 mRNA levels in the LPS-treated mouse placenta (**Figure 5C**). Immunohistochemical analysis showed that NLRP3 and IL-1β staining was significantly increased in the cytoplasm of trophoblasts in the junctional zone and labyrinth layer in LPS-treated mouse placenta; however, their expression was decreased with amlexanox treatment (**Figure 5D**).

**Figure 6:**
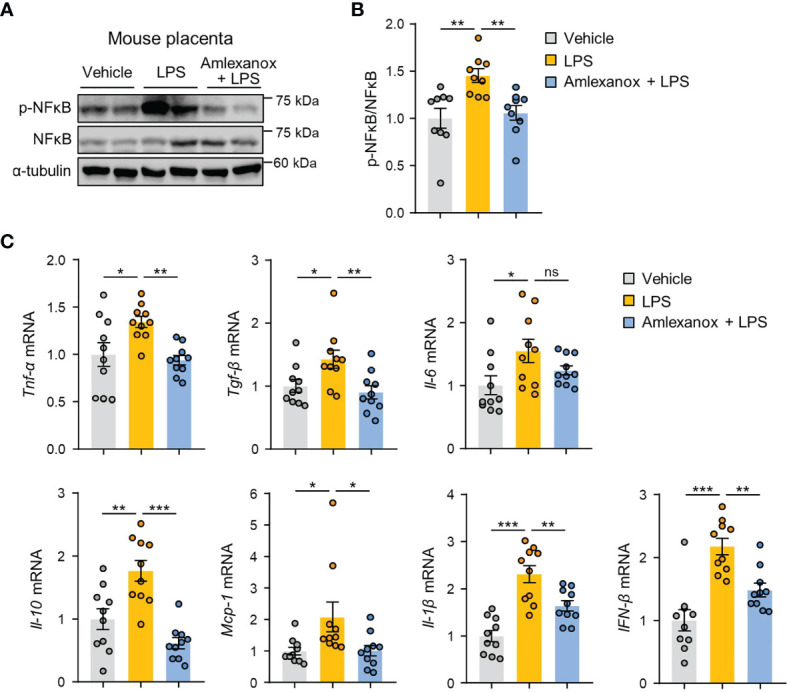
Amlexanox ameliorates LPS-induced inflammatory response in mouse placenta. **(A–C)** Pregnant mice were intraperitoneally injected with LPS (200 μg/kg) or amlexanox (2.5 mg/kg) on gestation day 17.5, as indicated in the figure. **(A, B)** Placental tissues were collected from mice and analyzed by immunoblotting with anti-p-p65 and anti-p65 antibodies (*n* = 9 per group). α-tubulin served as a loading control. Band intensities were quantified and normalized to control band intensities. **(C)** Placental tissues were collected from mice and analyzed by qRT-PCR for the expression of TNF-α, TGF-β, IL-6, IL-10, MCP-1, IL-1β, and IFN-β (*n* = 10 per group). Data are shown as means ± SEM. *p < 0.05; **p < 0.01; ***p < 0.001; ns, not significant (One-way ANOVA, followed by Fisher’s LSD test).

To determine whether the effect of amlexanox on the NF-κB pathway could be observed *in vivo*, we measured the phosphorylation levels of NF-κB/p65 in mouse placental tissues. Immunoblot analysis revealed that phosphorylation of NF-κB/p65 was increased, whereas amlexanox treatment prevented the increase in phosphorylation levels of NF-κB/p65 (**Figures 6A, B**). We also evaluated whether maternal administration of amlexanox affected gene expression of inflammatory cytokines and chemokines using qRT-PCR. The expression of IL-1β, IL-6, IL-10, TNF-α, TGF-β1, MCP-1, and IFN-β genes was elevated in the placenta of LPS-treated mice as compared to that of control mice; this increase was largely attenuated in amlexanox-treated mice exposed to LPS (**Figure 6C**). Taken together, these data indicate that TBK1 inhibition by amlexanox alleviates placental inflammation and NLRP3 inflammasome activation.

### TBK1 Deficiency Attenuates LPS-Induced NLRP3 Inflammasome Activation *via* an mTORC1-Dependent Mechanism

**Figure 7:**
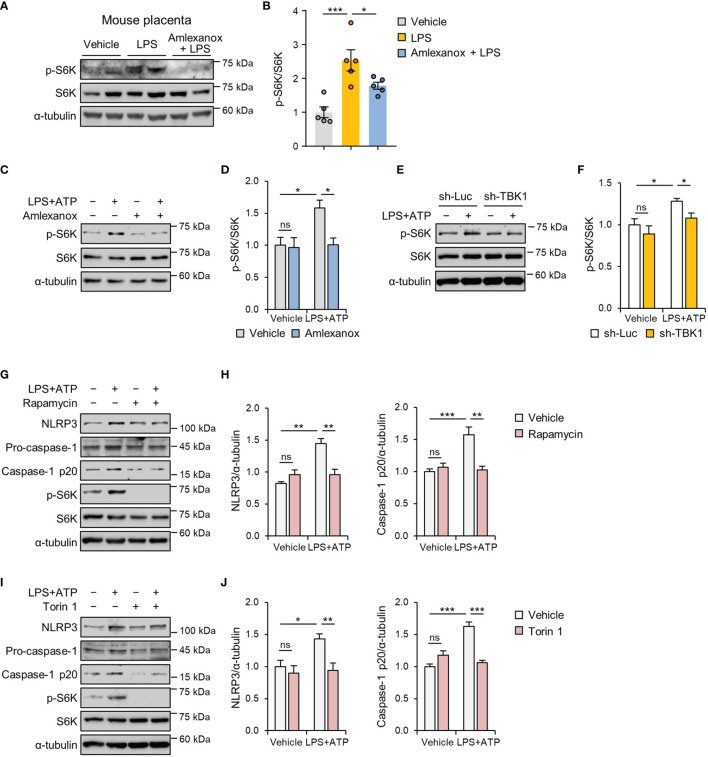
TBK1 deficiency suppresses LPS-induced NLRP3 inflammasome activation *via* an mTORC1-dependent mechanism. **(A, B)** Pregnant mice were intraperitoneally injected with LPS (200 μg/kg) or amlexanox (2.5 mg/kg) on gestation day 17.5, as indicated in the figure. Placental tissues were collected from mice and analyzed by immunoblotting with anti-p-S6K and anti-S6K antibodies (*n* = 5 per group). **(C, D)** HTR-8/SVneo cells were treated with 1 μg/mL LPS or 100 µM amlexanox for 24 h, as indicated in the figure. Cell lysates were analyzed by immunoblotting with anti-p-S6K and anti-S6K antibodies. α-tubulin served as a loading control. **(E, F)** HTR-8/SVneo cells were infected with lentiviruses expressing shRNAs targeting luciferase (sh-Luc) or TBK1 (sh-TBK1) and treated with 1 μg/mL LPS for 24 h, as indicated in the figure. Cell lysates were analyzed by immunoblotting with anti-p-S6K and anti-S6K antibodies. **(G–J)** HTR-8/SVneo cells were treated with 1 μg/mL LPS, 100 nM rapamycin, or 250 nM Torin 1 for 24 h, as indicated in the figure. Cell lysates were analyzed by immunoblotting with anti-NLRP3, anti-caspase-1, anti-p-S6K, and anti-S6K antibodies. α-tubulin served as a loading control. Band intensities were quantified and normalized to control band intensities. Data are shown as means ± SEM. Results are representative of at least three independent experiments. *p < 0.05; **p < 0.01; ***p < 0.001; ns, not significant (One-way ANOVA, followed by Tukey’s test).

In response to LPS, TBK1 can directly activate mTORC1, but this function of TBK1 has been controversial (29, 30). Therefore, we checked the expression of downstream target proteins of mTORC1 in the placental tissues of control, vehicle-, and amlexanox-treated LPS mice. Immunoblot analysis indicated that mice treated with amlexanox had lower levels of phosphorylated p70 ribosomal protein S6 kinase (p70S6K) compared to that in LPS-treated mice (**Figures 7A, B**). Further, we evaluated the effect of amlexanox on LPS-induced mTORC1 activation in HTR-8/SVneo cells and found a decrease in the phosphorylation of p70S6K after pretreatment with amlexanox (**Figures 7C, D**). Consistent with the inhibitory effect of amlexanox on mTORC1 signaling, lentiviral knockdown of TBK1 blocked LPS-induced phosphorylation of p70S6K in HTR-8/SVneo cells (**Figures 7E, F**), indicating that TBK1 is involved in LPS-mediated mTORC1 activation. To investigate whether mTORC1 inhibition influences LPS-induced NLRP3 inflammasome activation, we pretreated HTR-8/SVneo cells with mTOR inhibitors, rapamycin and Torin 1. Immunoblot analysis revealed suppression of LPS-induced increase in NLRP3 and cleaved caspase-1 protein levels after pretreatment with rapamycin or Torin 1 (**Figures 7G–J**). Thus, these data suggest that TBK1 deficiency alleviates LPS-induced NLRP3 inflammasome activation in an mTORC1-dependent manner.

## Discussion

**Figure 8:**
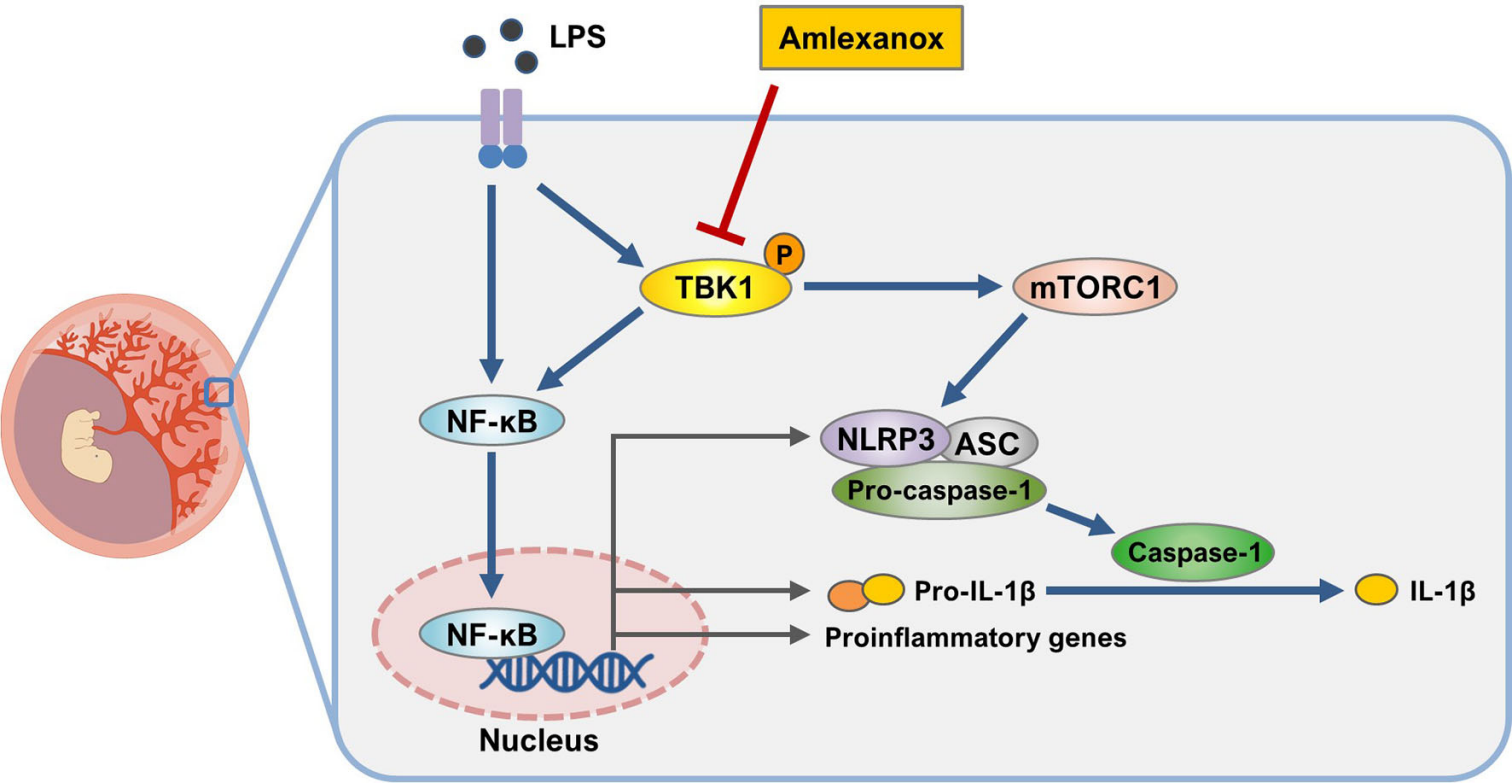
Schematic diagram of the role of TBK1 in trophoblasts upon exposure to LPS. Inhibition of TBK1 by amlexanox not only suppresses LPS-induced NLRP3 inflammasome activation but also attenuates inflammatory response by inhibiting mTORC1 signaling.

Excessive placental inflammation due to infectious and non-infectious stimuli is responsible for several adverse pregnancy outcomes, including preterm birth, intrauterine growth restriction, and preeclampsia. TBK1 is widely expressed in animals and is involved in the activation of the NF-κB pathway (31, 32). However, the regulation of TBK1 in the pathogenesis of pregnancy complications remains unclear. Here, we observed the phosphorylation of TBK1 during LPS-induced placental inflammation, as evident in mouse placental tissue and human trophoblasts. Elevated IL-1β production has been implicated in the pathogenesis of pregnancy syndromes associated with placental inflammation (33, 34). Growing evidence suggests that abnormal production of biologically active IL-1β is mediated by NLRP3 inflammasome activation in the maternal–placental interface trophoblasts (17, 26, 35, 36). Although experimental models have demonstrated that LPS stimulates the NLRP3 inflammasome in human trophoblasts, no studies have investigated the role of TBK1 in LPS-induced NLRP3 inflammasome activation. In this study, we demonstrated the molecular link between TBK1 phosphorylation and NLRP3 inflammasome activation in LPS-induced placental inflammation. Furthermore, TBK1 inhibition attenuates LPS-induced impairment of fetal and placental development, as assessed by fetal and placental weight and length (**Figure 8**), suggesting that TBK1 phosphorylation is involved in placental inflammation and NLRP3 inflammasome activation.

TBK1 is phosphorylated in adipose and liver tissues of obese mice (21, 37) and trophoblast cells in response to LPS stimulation (38), which agrees with our findings that LPS treatment induces TBK1 phosphorylation in mouse placental tissue and human trophoblasts. However, the relationship between LPS-associated placental inflammation and TBK1 activation had not been studied previously. A previous study from our group indicated that BX795, a TBK1 inhibitor, suppressed the expression of inflammatory cytokines, such as IL-6 and IL-10, in a nonalcoholic steatohepatitis mouse model; however, the relevant mechanism was not defined (21). Here, we found that maternal administration of amlexanox resulted in a decrease in the expression of inflammatory cytokine and chemokine genes, including IL-1β, IL-10, TNF-α, TGF-β1, MCP-1, and IFN-β in the placenta of LPS-treated mice. These effects are probably attributed to reduced TBK1 activity, which can regulate the expression of inflammatory mediators (39–41). IFN-β expression can be induced by pathogen-associated molecular patterns in trophoblast cells through TLR4/IRF3 signaling pathway (42–44). The role of IFN-β in pregnancy has been revealed recently. Kwon et al. reported that aberrant production of IFN-β and its associated IFN-stimulated gene activation result in fetal demise (38). Consistent with this finding, IFN-β gene expression was elevated in the placenta of LPS-treated mice as compared to that of control mice; this increase was largely attenuated in amlexanox-treated mice exposed to LPS. Although our current data clearly reveal a reduction in IL-1β expression following maternal administration of amlexanox in the placenta of LPS-treated mice, it is possible that blocking TBK1 activation might also affect IFN-β expression by activating IRF3. Given that TBK1 mediates phosphorylation and nuclear translocation of IRF3, which contributes to induction of type I IFNs (45, 46), our data suggest that the effect of TBK1 inhibition on the LPS-treated pregnant mice may be partially mediated through alteration of IFN-β production.

TBK1 contributes to the activation of the non-canonical NF-κB pathway (47–49). However, TBK1 may also inhibit the activation of the NF-κB pathway (50). Given that TBK1 is involved in cell- and stimulus-dependent NF-κB activation, we directly investigated the effect of TBK1 deficiency on the LPS-induced NF-κB pathway. Our results clearly demonstrated that the treatment of mice and human trophoblast cells with amlexanox reduced the LPS-induced NF-κB/p65 pathway. Thus, our data suggest that TBK1 inhibition is intimately involved in the negative regulation of the NF-κB/p65 pathway.

In conclusion, our results provide evidence for the potential role of TBK1 in LPS-induced activation of the placental NLRP3 inflammasome. LPS treatment upregulates the phosphorylation of TBK1, which mediates placental NLRP3 inflammasome activation and inflammation. Our study elucidates the TBK1-mTORC1 axis and its relevance to placental inflammation. Future investigation will be needed to explore the upstream signaling intermediates regulating the TBK1‐mTORC1 axis during LPS-induced placental inflammation. Our *in vitro* and *in vivo* study reveals a protective effect of pharmacological inhibition of TBK1 on LPS-induced NLRP3 inflammasome activation and inflammation, providing an interesting opportunity for repositioning of TBK inhibitors for pregnancy-related complications.

## Data Availability Statement

The raw data supporting the conclusions of this article will be made available by the authors, without undue reservation.

## Ethics Statement

The animal study was reviewed and approved by the Institutional Animal Care and Use Committee of Konyang University.

## Author Contributions

S-KL and H-WP: Conceptualization and experimental design. SL, JiS, JoS, and H-WP: Investigation and analysis. J-SK, JoS, and S-KL: Resources J-SK, JiS, and JoS: methodology. SL, JiS, and H-WP: Writing—Original draft preparation. S-KL and H-WP: Writing— review and editing. S-KL and H-WP: Verification and supervision. All authors contributed to the article and approved the submitted version.

## Funding

This research was supported by grants from the National Research Foundation of Korea funded by the Ministry of Science and ICT (No. 2015R1A5A1009701) and the Ministry of Education (No. NRF-2017R1A6A1A03015713). This research was also supported by a grant from the Korean Health Technology R&D Project funded by the Ministry of Health and Welfare, Republic of Korea (No. HI17C1238).

## Conflict of Interest

The authors declare that the research was conducted in the absence of any commercial or financial relationships that could be construed as a potential conflict of interest.

## Publisher’s Note

All claims expressed in this article are solely those of the authors and do not necessarily represent those of their affiliated organizations, or those of the publisher, the editors and the reviewers. Any product that may be evaluated in this article, or claim that may be made by its manufacturer, is not guaranteed or endorsed by the publisher.
