# Supplementary material for: Targeting TBK1 Attenuates LPS-Induced NLRP3 Inflammasome Activation by Regulating of mTORC1 Pathways in Trophoblasts
Source: Front Immunol. 2021 Nov 9;12:743700. doi: 10.3389/fimmu.2021.743700 (PMC8630692; doi:10.3389/fimmu.2021.743700)
Supplement: Supplementary file 1 [file DataSheet_1.docx]

SUPPLEMENTARY MATERIAL


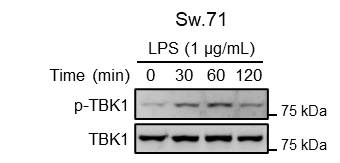


**Supplemental Figure 1.** Sw.71 cells were treated with LPS (1 μg/mL) for the indicated time periods. Cell lysates were immunoblotted with anti-p-TBK1 and anti-TBK1 antibodies. Total protein served as a loading control. Results are representative of at least three independent experiments.


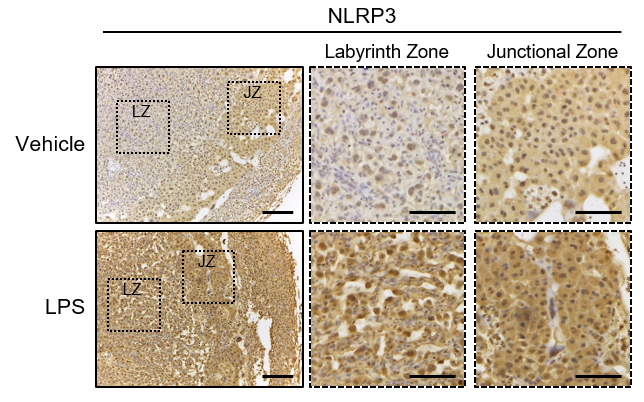


**Supplemental Figure 2.** Immunohistochemical analysis of NLRP3 in placental tissues from vehicle- and LPS -treated pregnant mice at gestation day 17.5. Nuclei were stained with hematoxylin. Boxed areas are magnified in the adjacent panels. LZ and JZ represent the labyrinth zone and the junctional zone, respectively. Scale bars, 200 μm; inset, 100 μm.


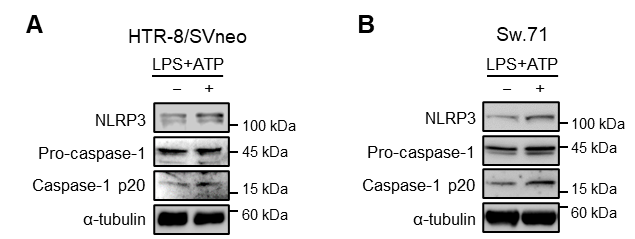


**Supplemental Figure 3.** (A, B) HTR-8/SVneo and Sw.71 cells were treated with 1 μg/mL LPS for 24 h followed by treatment with 5 mM ATP for 45 min. Cell lysates were analyzed by immunoblotting with anti-NLRP3 and anti-caspase-1 antibodies. α-tubulin served as a loading control. Results are representative of at least three independent experiments.


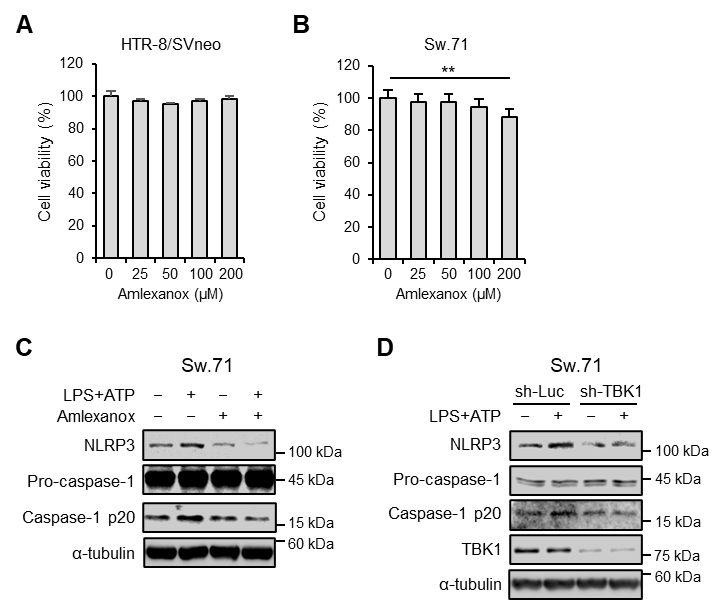


**Supplemental Figure 4.** (A, B) HTR-8/SVneo cells and Sw.71 cells were treated with the indicated concentration of amlexanox for 24 h. Cell viability was measured by WST-1 assay. (C) Sw.71 cells were treated with 1 μg/mL LPS or 100 µM amlexanox for 24 h followed by treatment with 5 mM ATP for 45 min, as indicated in the figure. Cell lysates were immunoblotted with anti-NLRP3 and anti-caspase-1 antibodies. (D) Sw.71 cells were infected with lentiviruses expressing shRNAs targeting luciferase (sh-Luc) or TBK1 (sh-TBK1) and treated with 1 μg/mL LPS for 24 h followed by treatment with 5 mM ATP for 45 min, as indicated in the figure. Cell lysates were immunoblotted with anti-NLRP3, anti-caspase-1, and anti-TBK1 antibodies. α-tubulin served as a loading control. Results are representative of at least three independent experiments.


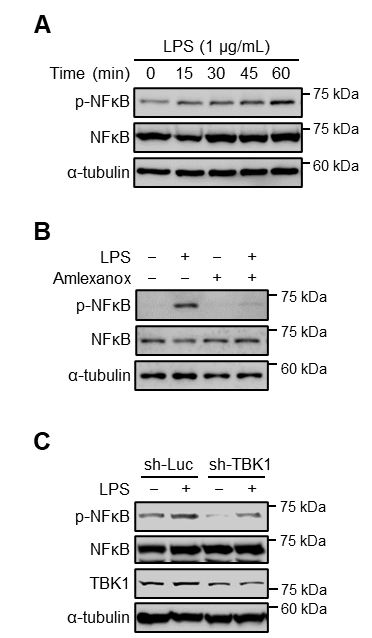


**Supplemental Figure 5.** (A) HTR-8/SVneo cells were treated with LPS (1 μg/mL) for the indicated time periods. Cell lysates were analyzed by immunoblotting with anti-p-p65 and anti-p65 antibodies. α-tubulin served as a loading control. (B) Sw.71 cells were treated with 1 μg/mL LPS or 100 µM amlexanox for 1 h, as indicated in the figure. Cell lysates were immunoblotted with anti-p-p65 and anti-p65 antibodies. (C) Sw.71 cells were infected with lentiviruses expressing shRNAs targeting luciferase (sh-Luc) or TBK1 (sh-TBK1) and treated with 1 μg/mL LPS for 1 h, as indicated in the figure. Cell lysates were immunoblotted with anti-p-p65 and anti-p65 antibodies. α-tubulin served as a loading control. Results are representative of at least three independent experiments.
